# Metabolically healthy and unhealthy obesity and risk of vasomotor symptoms in premenopausal women: cross‐sectional and cohort studies

**DOI:** 10.1111/1471-0528.17224

**Published:** 2022-06-07

**Authors:** Sunju Namgoung, Yoosoo Chang, Chae‐Yeon Woo, Yejin Kim, Jeonggyu Kang, Ria Kwon, Ga‐Young Lim, Hye Rin Choi, Kye‐Hyun Kim, Hoon Kim, Yun Soo Hong, Di Zhao, Juhee Cho, Eliseo Guallar, Hyun‐Young Park, Seungho Ryu

**Affiliations:** ^1^ Centre for Cohort Studies, Total Healthcare Centre, Kangbuk Samsung Hospital Sungkyunkwan University School of Medicine Seoul Korea; ^2^ Department of The Environmental Health Centre, Wonju Severance Christian Hospital Yonsei University School of Medicine Wonju Korea; ^3^ Department of Occupational and Environmental Medicine, Kangbuk Samsung Hospital Sungkyunkwan University School of Medicine Seoul Korea; ^4^ Institute of Medical Research Sungkyunkwan University, School of Medicine Suwon Republic of Korea; ^5^ Department of Obstetrics and Gynaecology, Kangbuk Samsung Hospital Sungkyunkwan University School of Medicine Seoul Korea; ^6^ Department of Obstetrics and Gynaecology Seoul National University College of Medicine Seoul Korea; ^7^ Departments of Epidemiology and Medicine, and Welch Center for Prevention, Epidemiology, and Clinical Research Johns Hopkins University Bloomberg School of Public Health Baltimore Maryland USA; ^8^ Department of Clinical Research Design & Evaluation, SAIHST Sungkyunkwan University Seoul Korea; ^9^ Department of Precision Medicine National Institute of Health, Korea Disease Control and Prevention Agency (KDCA) Cheongju Korea

**Keywords:** body composition, body mass index, cohort study, metabolic health, obesity, percentage body fat, premenopausal women, vasomotor symptoms, waist circumference

## Abstract

**Objective:**

To examine the relationship between metabolically healthy and unhealthy obesity phenotypes and risk of vasomotor symptoms (VMS) in premenopausal women.

**Design:**

Prospective cohort study.

**Setting:**

Middle‐aged women in a cohort based on regular health screening examinations.

**Population:**

Premenopausal Korean women aged 42–52 years were recruited and were followed up for a median of 4.2 years. The cross‐sectional and cohort studies comprised 4672 women and 2590 women without VMS at baseline, respectively.

**Methods:**

Adiposity measures included body mass index (BMI), waist circumference and percentage body fat. Being metabolically healthy was defined as not having any metabolic syndrome components or a homeostasis model assessment of insulin resistance of 2.5 or more.

**Main outcomes measures:**

VMS (hot flushes and night sweats) assessed using the questionnaire.

**Results:**

All adiposity measures were positively associated with an increased risk of VMS in both cross‐sectional and longitudinal studies. The multivariable‐adjusted prevalence ratio (95% confidence interval [CI]) for VMS comparing percentage body fat of 35% or more with the reference was 1.47 (95% CI 1.14–1.90) in metabolically healthy women, and the corresponding prevalence ratio was 2.32 (95% CI 1.42–3.78) in metabolically unhealthy women (*P*_interaction_ = 0.334). The multivariable‐adjusted hazard ratio for incident VMS comparing percentage body fat of 35% or more with the reference was 1.34 (95% CI 1.00–1.79) in metabolically healthy women, whereas the corresponding hazard ratio was 3.61 (95% CI 1.81–7.20) in metabolically unhealthy women (*P*_interaction_ = 0.036). The association between BMI, waist circumference and VMS did not significantly differ by metabolic health status.

**Conclusions:**

Maintaining normal weight and being metabolically healthy may help to prevent VMS in premenopausal women.

**Tweetable abstract:**

Avoiding obesity and a metabolically unhealthy status may help reduce vasomotor symptoms in premenopausal women.

## Tweetable abstract

Avoiding obesity and a metabolically unhealthy status may help reduce vasomotor symptoms in premenopausal women.

## 1 INTRODUCTION

Vasomotor symptoms (VMS), including hot flushes and night sweats, are cardinal symptoms of menopause that affect 60–80% of climacteric women and substantially reduce their quality of life.^1^, ^2^, ^3^, ^4^ Contrary to the previous belief that VMS occur near the final menstrual period and are short lived (6 months to 2 years), recent studies have demonstrated that VMS are commonly observed in the premenopausal and early menopausal transition stages and may persist long after menopause.^5^, ^6^, ^7^ Furthermore, early onset and long duration of VMS were associated with adverse psychosocial and physical profiles and increased risk of subclinical and cardiovascular disease.^8^, ^9^, ^10^ Despite their impact on the quality of life and risk profile of women, few studies have evaluated the distinctive characteristics of early‐onset VMS.^5^

Overweight and obesity are associated with VMS,^2^, ^11^, ^12^, ^13^ and weight loss may reduce VMS.^14^ These findings support the notion that obesity is a risk factor for VMS. Obesity is frequently accompanied by metabolic abnormalities such as type 2 diabetes, hypertension, dyslipidaemia and insulin resistance, but a subset of obese individuals do not present metabolic abnormalities despite having excessive body fat, a phenomenon referred to as metabolically healthy obesity.^15^ No study has examined the role of metabolically healthy obesity on VMS risk. In this study, we aimed to test the hypothesis that metabolically healthy and unhealthy obesity phenotypes affect VMS differently using cross‐sectional and longitudinal studies of premenopausal women.

## 2 METHODS

### 2.1 Study population

For a longitudinal study of midlife Korean women, participants were recruited between 2014 and 2018 from the Kanbuk Samsung Health Study, a cohort study of Korean adults who received annual or biennial comprehensive health examinations at the clinics of Kangbuk Samsung Hospital Total Healthcare Centres in Seoul and Suwon, South Korea. The eligibility criteria for enrolment included (1) age 42–52 years; (2) no history of hysterectomy, oophorectomy or hormone replacement therapy; (3) at least one menstrual period in the 3 months before the health screening examination and no amenorrhea lasting for 60 days or more; and (4) no history of a chronic disease that may affect menstrual cycles (malignancy, renal failure and hypo‐ or hyperthyroidism). For the cross‐section study, exclusion criteria included study withdrawal and missing information on VMS or adiposity measures or metabolic component (Figure S1). Next, the exclusion criteria for the cohort study were (1) prevalent VMS at baseline; (2) no follow up; and (3) missing information on VMS.

### 2.2 Ethical considerations

The present study was approved by the institutional review board of Kangbuk Samsung Hospital (IRB No. KBSMC 2021–01‐054). Written consent was obtained from all study participants after they were fully informed of the purpose of the study, and all the procedures were conducted according to all applicable institutional and governmental regulations concerning the ethical use of human volunteers in compliance with the Declaration of Helsinki.

### 2.3 Measurements

Information regarding demographic characteristics, lifestyle factors, reproductive factors and medication use was obtained through a standardised, structured, self‐administered questionnaire. Smoking status was categorised into never smoking, former smoking and current smoking. Average alcohol intake was categorised into none, up to 10 g/day, more than 10 g/day or unknown.^9^ Physical activity was assessed using the validated Korean version of the International Physical Activity Questionnaire short form and categorised into inactive, minimally active and health‐enhancing physical activity.^16^ Education level was categorised as less than college graduate versus college graduate or higher. Parity, as a reproductive factor, was defined as the number of previous pregnancies, including live births and stillbirths.

Menopausal stages were categorised based on the criteria of the Stages of Reproductive Aging Workshop (STRAW) +10 as: (1) premenopause; (2) early menopausal transition, defined as a persistent difference of 7 or more days in length of consecutive cycles; (3) late menopausal transition, defined as amenorrhea of 60 or more days; (4) postmenopause, defined as amenorrhea of 12 or more months.^17^

Height, weight and body composition were measured by trained nurses, with the participants wearing a lightweight hospital gown and no shoes. The percentage of body fat was estimated using a multifrequency bioimpedance analyser with eight‐point tactile electrodes (InBody 720; Biospace Co., Seoul, Korea), validated regarding reproducibility and accuracy for body composition.^18^ Waist circumference was measured by trained personnel to the nearest 0.1 cm at the midpoint between the bottom of the rib cage and the top of the iliac crest with the participants standing with their weight equally distributed on both feet, their arms at their sides and head facing straight forward. Hypertension was defined as systolic blood pressure (BP) at least 140 mmHg, diastolic BP at least 80 mmHg, or current use of antihypertensive medication.

Blood samples were taken from the antecubital vein after at least a 10‐hour fast and were measured for glucose, glycated hemoglobin, insulin and serum lipid profiles, including total cholesterol, low‐density lipoprotein cholesterol, high‐density lipoprotein cholesterol and triglycerides. Diabetes mellitus was defined as fasting serum glucose at least 126 mg/dL, glycated hemoglobin at least 6.5% (48 mmol/mol), or current use of insulin or glucose‐lowering medication. Insulin resistance was assessed using the homeostatic model assessment of insulin resistance according to the following formula: fasting blood insulin (mU/mL) × fasting blood glucose (mmol/L)/22.5.

### 2.4 Metabolically healthy and unhealthy obesity

Body mass index (BMI) was categorised according to Asian‐specific criteria^19^ as underweight (BMI <18.5 kg/m^2^), normal weight (BMI 18.5–22.9 kg/m^2^), overweight (BMI 23–24.9 kg/m^2^), obesity I (BMI 25–29.9 kg/m^2^) and obesity II (BMI ≥30 kg/m^2^). Only a small proportion (5.3%) of study participants were categorised as underweight and they were combined into the normal weight category. The proportion of obesity II (BMI ≥30 kg/m^2^) was less than 2% and obesity I and II were combined into a single obesity category. Abdominal obesity was defined as waist circumference of 80 cm or more, also a specific criterion for the Asian population.^20^ Percentage body fat was categorised as less than 25.0%, 25.0%–29.9%, 30.0%–34.9% and 35.0% or more.

Metabolically unhealthy persons were defined as those having at least one of the following metabolic abnormalities: fasting glucose level 100 mg/dL or more or current use of glucose‐lowering agents; BP at least 130/85 mmHg or current use of BP‐lowering agents; triglyceride level at least 150 mg/dL or current use of lipid‐lowering agents; high‐density lipoprotein cholesterol level less than 50 mg/dL; or insulin resistance, defined as homeostatic model assessment of insulin resistance score at least 2.5.^21^ Being metabolically healthy was defined as having none of the metabolic abnormalities described above.^22^

### 2.5 Prevalent and incident VMS

At baseline and at each follow‐up visit, we used the Korean version of the Menopause‐Specific Quality of Life questionnaire^23^, ^24^ to assess the presence and bothersome degree of VMS, including hot flushes and night sweats. If the participant responded ‘No’ to hot flushes or night sweats, she was considered as not having VMS. If the participant responded ‘Yes’ and experienced hot flushes or night sweats and rated them on the bothered scale, she was considered as having VMS. Prevalent VMS were defined as VMS present at baseline, whereas incident VMS were defined as the new onset of VMS during follow up among participants free of VMS at baseline.

### 2.6 Statistical analyses

The characteristics of the study participants are presented according to the study design using descriptive summary statistics. For the cross‐sectional study, Poisson regression models with robust variance were used to estimate prevalence ratios and 95% confidence intervals (CIs) for VMS at baseline according to adiposity parameters, including BMI, waist circumference and percentage body fat. Poisson regression models are most frequently applied to estimate risk ratios for common binary outcomes.^25^, ^26^ We applied robust standard errors to address variance overestimation when Poisson regression was applied to binary data.^26^

For the longitudinal cohort analysis, the primary outcome was incident VMS before menopause. Each participant was followed from the baseline examination until the time of VMS occurrence, the time of menopause, or the last questionnaire survey date (4 October 2020), whichever was first. The new‐onset VMS was assessed based on the Menopause‐Specific Quality of Life, wherein the time frame is ‘in the Past month’, thus limiting exact estimations of VMS onset time as the study participants visited and completed the questionnaire every 1 or 2 years during the median follow‐up duration of 4.2 years (interquartile range 3.1–5.1 years). Because the occurrence of VMS was known to have occurred between the two visits (visit with first report of VMS and the previous visit), but the exact time at which it developed was unknown, a parametric proportional hazards model was used to account for this type of interval censoring (stpm command in stata)^27^ and to estimate adjusted hazard ratios and 95% CIs for new‐onset VMS according to obesity phenotype. In these models, the baseline hazard function was parameterised with restricted cubic splines in log time with four degrees of freedom.

For adjustment of covariates, the model was first adjusted for age, and then further adjusted for menarche age, educational attainment, parity (nulliparous or parous), physical activity, smoking status and alcohol intake. To test for linear trends, the number of categories was treated as a continuous variable. We also performed stratified analyses based on metabolic health status. Likelihood ratio tests comparing models with and without multiplicative interaction terms were used to evaluate interactions by metabolic health status. Furthermore, we examined the relationship between adiposity measures as a continuous variable and VMS risk. For this analysis, we modelled the adiposity measures as restricted cubic splines with knots at the 5th, 27.5th, 50th, 72.5th and 95th centiles of the sample distribution to provide a flexible estimate of the concentration–response relationship between adiposity measures and VMS risk.

Statistical analyses were performed using STATA version 16.0 (Stata Corp LP; College Station, TX, USA). Two‐sided *P* values less than 0.05 were considered statically significant.

## 3 RESULTS

### 3.1 Obesity phenotypes and prevalent VMS

Of 5230 participants initially enrolled, we excluded 194 women who withdrew from participation and 81 women with missing information on VMS or adiposity measures or metabolic components, leaving 4672 participants in the cross‐sectional study (Figure S1). In the cross‐sectional analysis, the mean age of 4672 participants was 44.9 ± 2.5 years and the VMS prevalence was 18.8% (Table 1). The prevalence of obesity (BMI ≥25 kg/m^2^), abdominal obesity (waist circumference ≥80 cm) and high percentage body fat of 35% or more were 16.9, 28.0 and 21.2%, respectively. The prevalence of being metabolically unhealthy was 32.3%. Women with prevalent VMS were more likely to be older and have higher adiposity measures and unfavourable profiles of metabolic components than those without (Table S1).

**Table 1:** Baseline characteristics of study participants by study design

Characteristics	Cross‐sectional study (*n* = 4672)	Longitudinal study (*n* = 2525)
Age (years)	44.9 ± 2.5	44.7 ± 2.4
Prevalent case of VMS (%)	18.8	
Being metabolically unhealthy (%)	32.3	29.9
Number of metabolic abnormalities		
0	67.7	70.1
1	19.5	18.7
2	7.9	6.9
≥3	4.9	4.2
BMI category (kg/m^2^)		
<22.9	63.7	66.1
23.0–24.9	19.5	18.1
25.0–29.9	14.9	14.0
≥30	2.0	1.8
Waist circumference ≥ 80 cm (%)	28.0	26.0
Percentage body fat (%)		
<25.0	16.7	17.7
25.0–29.9	29.8	30.9
30.0–34.9	32.3	32.0
≥35.0	21.2	19.5
Age at menarche (years)	14.0 ± 1.4	13.9 ± 1.4
Parity (%)	92.0	92.3
Ever smoker (%)	11.7	11.3
Alcohol intake (%)^a^	13.0	11.5
HEPA (%)	15.5	15.3
Higher education (%)^b^	80.1	81.7
Hypertension (%)	5. 0	3.9
Diabetes (%)	2.1	1.9
Medication for hyperlipidaemia (%)	1.6	1.6
Systolic BP (mmHg)	103.9 ± 11.7	103.2 ± 11.3
Diastolic BP (mmHg)	66.9 ± 9.2	66.4 ± 8.7
Glucose (mg/dL)	93.2 ± 12.5	92.6 ± 11.1
LDL‐C (mg/dL)	119.0 ± 28.8	118.0 ± 28.4
HDL‐C (mg/dL)	66.7 ± 15.9	67.4 ± 16.0
Triglycerides (mg/dL)	75 (58–101)	73 (56–98)
HOMA‐IR	1.1 (0.8–1.6)	1.1 (0.8–1.6)
hsCRP (mg/L)^c^	0.3 (0.2–0.6)	0.3 (0.2–0.6)

Data presented are mean ± standard deviation, median (interquartile range), or percentage.

Abbreviations: ALT, alanine aminotransferase; BMI, body mass index; BP, blood pressure; HDL‐C, high‐density lipoprotein cholesterol; HEPA, health‐enhancing physical activity; HOMA‐IR, homeostasis model assessment of insulin resistance; hsCRP, high‐sensitivity C‐reactive protein; LDL‐C, low‐density lipoprotein cholesterol; VMS, vasomotor symptoms.

^a^ At least 10 g of ethanol per day.

^b^ College graduate or beyond.

^c^ Among 3056 participants (cross‐sectional study) and 1727 (longitudinal study).

Increased adiposity measures were significantly associated with a higher prevalence of VMS in a dose‐dependent manner (Table 2). The multivariable‐adjusted prevalence ratios for VMS comparing overweight and obesity to normal weight were 1.24 (95% CI 1.07–1.44) and 1.42 (95% CI 1.23–1.64), respectively (*P* for trend <0.001). Similarly, the multivariable‐adjusted prevalence ratios comparing percentage body fat 25%–29.9%, 30%–34.9% and at least 35% with percentage body fat less than 25% as the reference category were 1.18 (95% CI 0.96–1.46), 1.35 (95% CI 1.10–1.66) and 1.73 (95% CI 1.41–2.13), respectively (*P* for trend <0.001). For waist circumference, the multivariable‐adjusted prevalence ratio for VMS comparing abdominal obesity to the normal group was 1.29 (95% CI 1.14–1.47). Being metabolically unhealthy was associated with increased prevalence of VMS with corresponding prevalence ratio of 1.39 (95% CI 1.23–1.57). In spline regression models, BMI and waist circumference exhibited a J‐shaped association with VMS prevalence, whereas the VMS prevalence linearly increased across the range of fat percentages (Figure S2).

**Table 2:** Cross‐sectional association between adiposity measures and prevalence of vasomotor symptoms among premenopausal women (*n* = 4672)

Obesity type	No. of cases	Prevalence (%)	Age‐adjusted PR (95% CI)	Multivariable‐adjusted PR (95% CI)
BMI category (kg/m^2^)				
<23.0	496	16.7	Reference	Reference
23.0–24.9	192	21.1	1.24 (1.07–1.45)	1.24 (1.07–1.44)
≥25	192	24.5	1.44 (1.24–1.67)	1.42 (1.23–1.64)
*P* for trend			<0.001	<0.001
Waist circumference (cm)				
<80	580	17.2	Reference	Reference
≥80	300	22.9	1.31 (1.16–1.48)	1.29 (1.14–1.47)
Percentage body fat (%)				
<25.0	108	13.9	Reference	Reference
25.0–29.9	233	16.8	1.20 (0.97–1.48)	1.18 (0.96–1.46)
30.0–34.9	291	19.3	1.37 (1.11–1.67)	1.35 (1.10–1.66)
≥35.0	248	25.0	1.77 (1.44–2.18)	1.73 (1.41–2.13)
*P* for trend			<0.001	<0.001
Metabolic health status				
Healthy	522	16.5	Reference	Reference
Unhealthy	358	23.7	1.41 (1.25–1.59)	1.39 (1.23–1.57)

Poisson regression models with robust variance were used to estimate PRs and 95% CIs for prevalent vasomotor symptoms. The multivariable model was adjusted for age, education level, parity, physical activity, smoking status and alcohol intake.

Abbreviations: BMI, body mass index; CI, confidence interval; PR, prevalence ratio.

### 3.2 Obesity phenotypes and incident VMS

The cohort study was restricted to women free of VMS at baseline who had at least one follow‐up visit. We therefore excluded 880 women who reported VMS at baseline and 1264 women with no follow‐up visit, as well as three women with missing information on VMS. The final sample for the cohort study included 2525 participants (Figure S1). At baseline, the mean ± standard deviation age of participants was 44.7 ± 2.4 years (Table 1). At baseline, women who developed VMS were more likely to be older, metabolically unhealthy and have higher adiposity measures, higher systolic BP and unfavorable lipid profiles than those who did not (Table S2).

During 10 087.4 person‐years of follow up, we identified 832 cases of incident VMS (incidence rate of 8.2 per 100 person‐years). The median follow‐up duration was 4.2 years (interquartile range 3.1–5.1 years). Higher levels of all adiposity measures were significantly associated with an increased risk of incident VMS. The multivariable‐adjusted hazard ratios comparing percentage body fat 25%–29.9%, 30%–34.9% and 35% or more with percentage body fat less than 25% as the reference category were 1.22 (95% CI 0.98–1.54), 1.42 (95% CI 1.14–1.76) and 1.60 (95% CI 1.26–2.03), respectively (*P* for trend <0.001). Similarly, BMI showed a dose–response relationship with an increasing risk of incident VMS (*P* for trend = 0.001). Abdominal obesity was also significantly associated with increased risk of incident VMS. Being metabolically unhealthy tended to show increased risk of VMS but this association did not reach statistical significance (Table 3). In spline regression models, the risk of incident VMS increased until approximately 25 kg/m^2^ without any further increase in VMS risk, whereas the VMS incidence linearly increased across the range of fat percentages (Figure S3).

**Table 3:** Longitudinal association between adiposity measures and incidence of vasomotor symptoms among premenopausal women free of vasomotor symptoms at baseline (*n* = 2525)

Obesity type	Person‐years	Early‐onset VMS	Incidence rate (cases per 100 PY)	Age‐adjusted HR (95% CI)	Multivariable‐adjusted HR (95% CI)
BMI category (kg/m^2^)					
<23.0	6700.4	495	7.4	Reference	Reference
23.0–24.9	1823.3	179	9.8	1.31 (1.11–1.56)	1.29 (1.09–1.54)
≥25	1563.8	158	10.1	1.32 (1.10–1.58)	1.31 (1.09–1.58)
*P* for trend				<0.001	0.001
Waist circumference (cm)					
<80	7501.8	574	7.7	Reference	Reference
≥80	2585.7	258	10.0	1.28 (1.11–1.49)	1.28 (1.11–1.49)
Percentage body fat (%)					
<25.0	1789.5	113	6.3	Reference	Reference
25.0–29.9	3160.9	244	7.7	1.22 (0.97–1.52)	1.22 (0.98–1.54)
30.0–34.9	3235.8	289	8.9	1.42 (1.14–1.76)	1.42 (1.14–1.76)
≥35.0	1901. 2	186	9.8	1.56 (1.23–1.97)	1.60 (1.26–2.03)
*P* for trend				<0.001	<0.001
Metabolic health status					
Healthy	7094.9	561	7.9	Reference	Reference
Unhealthy	2992.6	271	9.1	1.11 (0.96–1.29)	1.11 (0.96–1.28)

Parametric proportional hazard models were used. The multivariable model was adjusted for age, educational level, parity, physical activity, smoking status and alcohol intake.

Abbreviations: BMI, body mass index; CI, confidence interval; HR, hazard ratio; PY, person‐years; VMS, vasomotor symptoms.

When we compared the characteristics of participants with successful follow up and those lost to follow up, those lost to follow up were more likely to be older (Table S3). To control for the possibility of selection bias, we performed analyses using inverse probability weights based on the determinants of having a follow‐up visit over the study period. The results obtained using inverse probability weights were very similar to those obtained without inverse probability weights (Table S4). In analyses without restriction before menopause during follow up, the results were similar (Table S5).

### 3.3 Metabolically healthy and unhealthy obesity phenotypes and VMS risk

The positive associations between increased adiposity measures and the prevalence of VMS did not differ according to metabolic health status (cross‐sectional analysis, Table 4). Although the association between percentage body fat and prevalent VMS tended to be stronger in metabolically unhealthy participants, the interaction did not reach statistical significance (*P* for interaction = 0.334). Specifically, the multivariable‐adjusted prevalence ratio comparing percentage body fat of at least 35% with the reference category was 1.47 (95% CI 1.14–1.90) in metabolically healthy women and 2.32 (95% CI 1.42–3.78) in metabolically unhealthy women.

**Table 4:** Cross‐sectional and longitudinal associations between adiposity measures and VMS among metabolically healthy and unhealthy premenopausal women

Adiposity measures	Cross‐sectional analysis (*n* = 4672) Multivariable‐adjusted prevalence ratios (95% CI)^a^			Longitudinal analysis (*n* = 2525) Multivariable‐adjusted hazard ratios (95% CI)^b^		
	Metabolically healthy (*n* = 3162)	Metabolically unhealthy (*n* = 1510)	*P* for interaction	Metabolically healthy (*n* = 1771)	Metabolically unhealthy (*n* = 754)	*P* for interaction
BMI category (kg/m^2^)						
<22.9	Reference	Reference	0.775	Reference	Reference	0.449
23.0–24.9	1.16 (0.95–1.42)	1.23 (0.98–1.55)		1.26 (1.02–1.56)	1.38 (1.00–1.90)	
≥25	1.34 (1.05–1.70)	1.25 (1.01–1.55)		1.25 (0.94–1.66)	1.35 (1.02–1.80)	
*P* for trend	0.007	0.035		0.026	0.032	
Waist circumference (cm)						
<80	Reference	Reference	0.751	Reference	Reference	0.799
≥80	1.22 (1.01–1.46)	1.18 (0.98–1.41)		1.29 (1.05–1.59)	1.22 (0.95–1.56)	
Percentage body fat (%)						
<25.0	Reference	Reference	0.334	Reference	Reference	0.036
25.0–29.9	1.04 (0.82–1.31)	1.81 (1.09–3.01)		1.05 (0.82–1.34)	3.05 (1.50–6.16)	
30.0–34.9	1.18 (0.94–1.49)	1.90 (1.16–3.11)		1.31 (1.03–1.66)	2.78 (1.39–5.56)	
≥35.0	1.47 (1.14–1.90)	2.32 (1.42–3.78)		1.34 (1.00–1.79)	3.61 (1.81–7.20)	
*P* for trend	0.002	<0.001		0.008	0.001	

Abbreviations: BMI, body mass index; CI, confidence interval; HR, hazard ratio; VMS, vasomotor symptoms.

^a^ Poisson regression models with robust variance were used to estimate the prevalence ratios with 95% confidence intervals.

^b^ Parametric proportional hazard models were used to estimate the hazard ratios with 95% confidence intervals. The multivariable model was adjusted for age, education level, parity, physical activity, smoking status and alcohol intake.

On the other hand, the association of percentage body fat with incident VMS was stronger in the metabolically unhealthy group (longitudinal analysis, Table 4). The multivariable‐adjusted hazard ratio (95% CI) for incident VMS comparing percentage body fat of at least 35% to the reference category was 1.34 (95% CI 1.00–1.79) in metabolically healthy women and 3.61 (95% CI 1.81–7.20) in metabolically unhealthy women (*P* for interaction = 0.036). In analyses without restriction before menopause during follow up, the results were similar (Table S6).

In analyses stratified by education level (less than college graduate versus college graduate or more) (Table S7), the association between adiposity measures and VMS was similarly observed in both education categories without a significant interaction by education level.

## 4 DISCUSSION

### 4.1 Main findings

In this cross‐sectional and longitudinal analysis of midlife Korean women, BMI, waist circumference and percentage body fat were associated with an increased risk of VMS in premenopausal women. In the cross‐sectional analysis, we observed a positive association between obesity, abdominal obesity, percentage body fat and VMS prevalence in both metabolically healthy and unhealthy women with no significant interaction by metabolic health status. In the cohort analysis, the association of percentage body fat with incident VMS was stronger in the metabolically unhealthy group with significant interaction by metabolic status.

### 4.2 Interpretation

Previous studies and a meta‐analysis have demonstrated that obesity is associated with an increased risk of VMS.^12^ Although the role of obesity in VMS is well documented, the association of obesity phenotypes based on metabolic health status and body composition with VMS has not been investigated in detail. Women with higher adiposity show higher estrogen concentrations than lean women, which could potentially counteract the effect of estrogen deficiency during perimenopause and therefore lower the likelihood of VMS.^28^, ^29^ However, we found that obesity phenotypes as defined by BMI, waist circumference and percentage body fat were associated with incident VMS, which is in line with recent studies.^11^, ^30^ Several studies reported that women with metabolic abnormalities had a higher risk of prevalent VMS than those without,^31^, ^32^ but these studies did not specifically incorporate obesity phenotypes based on metabolic health, making it difficult to understand the role of obesity per se with or without metabolic abnormalities on VMS.

The mechanisms by which adiposity increases the risk of VMS in premenopausal women have not been fully established. Increased fat content may interfere with body heat dissipation, thereby increasing the risk of VMS.^33^ In a subcohort of the SWAN study, an adverse adipokine profile, which is also linked to the metabolic syndrome and obesity, was associated with a greater risk of VMS, especially in the early stage of menopausal transition.^11^ VMS is also related to changes in endogenous sex hormone levels, such as lower estradiol and higher follicle‐stimulating hormone, which are affected by adiposity and lipids.^34^, ^35^ Estrogen is known to regulate insulin receptor expression on peripheral tissues, especially on adipocytes, and is also involved in pathways associated with other cardio‐metabolic dysfunction such as dyslipidaemia, hypertension and type 2 diabetes.^36^ Given that lower estrogen levels are associated with a higher likelihood of developing VMS,^1^ it is possible that an interplay between reduced estrogen levels during perimenopause and existing metabolic abnormalities may synergistically influence and exacerbate outcomes in metabolically unhealthy women. Also, adipose tissue is an active endocrine organ that secretes multiple cytokines and inflammatory factors that may lead to VMS and ovarian dysfunction.^37^, ^38^ Adipose tissue produces leptin and tumour necrosis factor‐α, which influence thermoregulation.^39^, ^40^ As a result, a minor change in core body temperature may cause a series of neuroendocrine responses to maintain core temperature, ultimately leading to vasomotor flushing as a compensatory vascular response.^41^ Evidence suggests that pro‐inflammatory adipokine levels were significantly higher in metabolically unhealthy obesity compared with metabolically healthy obesity.^42^ VMS are also associated with over‐activity of sympathetic nervous system, and sympathetic overdrive seen in metabolic abnormalities may partially explain the association between metabolically unhealthy phenotypes and VMS risk.^43^, ^44^ Likewise, excess adiposity and its related adverse metabolic milieu might synergistically increase the risk of VMS.

Among adiposity measures, body fat percentage was most strongly associated with an increased risk of VMS. The stronger association based on body fat percentage could be related to the smaller amount of measurement error in body fat percentage compared with BMI: BMI is an indirect measure of body fat, with inherent limitations owing to its inability to differentiate fat mass from muscle mass;^45^, ^46^ waist circumference is also an indirect measure of visceral adiposity, which does not differentiate subcutaneous from visceral fat deposition, and also shows relatively large measurement errors.^47^ However, the differences across adiposity measures might be explained by chance because of multiple comparisons. Further studies with more accurate measures and a larger sample size are necessary to confirm our findings in diverse populations.

### 4.3 Strengths and limitations

Our study had several limitations. First, we used adiposity measures at baseline and did not consider changes in obesity status during follow up despite its dynamic nature over time. Second, confounders including smoking status and alcohol intake were assessed using self‐reported questionnaires, which may lead to misclassification. Additionally, we cannot exclude unmeasured confounders. Third, our study cohort was not population‐based and was relatively highly educated compared with the general population.^48^ In subgroup analyses by education level, the main findings were similar in both education categories with no significant interaction. Additionally, we used an Asian‐specific BMI cutoff for obesity based on the accumulative evidence that Asians have a higher total body fat and higher risk of metabolic and cardiovascular disease compared with Caucasians given the same level of BMI.^19^, ^49^ As a result, our study findings in midlife Korean women may not be readily generalisable and should be confirmed in other populations with different race/ethnicity compositions and demographic characteristics. The strengths of our study include the prospective design, the large sample size of a well‐characterised population of perimenopausal women and the use of carefully standardised clinical, lifestyle and laboratory measures, which allowed us to account for multiple potential confounders.

## CONCLUSIONS

In this cross‐sectional and longitudinal analysis, BMI, waist circumference and percentage body fat were positively associated with VMS in premenopausal women, particularly among metabolically unhealthy women with a high percentage body fat. Our findings suggest that maintaining both normal weight and being metabolically healthy may help reduce the risk of VMS in premenopausal women.

## AUTHORS CONTRIBUTIONS

Conception and design of study: Sunju Namgoung, Yoosoo Chang, Seungho Ryu. Acquisition of data: Min‐Jung Kwon, Ria Kwon, Ga‐Young Lim, Hyun‐Young Park. Analysis and /or interpretation of data: Sunju Namgoung, Yoosoo Chang, Seungho Ryu, Yejin Kim, Ria Kwon, Ga‐Young Lim, Chae‐Yeon Woo, Juhee Cho, Yun Soo Hong, Di Zhao, Jeonggyu Kang, Kye‐Hyun Kim, Hoon Kim, Hyun‐Young Park, Eliseo Guallar. Drafting the manuscript: SunJu NamGoung, Yoosoo Chang. Revising the manuscript critically for important intellectual content: all authors. Approval of the version of the manuscript to be published: all authors.

## CONFLICT OF INTERESTs

All authors declare no conflicts of interest.

## ETHICAL APPROVAL

This study was ethically approved by the institutional review board of Kangbuk Samsung Hospital (IRB No. KBSMC 2021–01‐054).

## Supporting information


Appendix S1

Data S1

Data S2

Data S3

Data S4

Data S5

Data S6

Data S7

Data S8

Data S9

Data S10

Data S11

Data S12

Data S13

Data S14

Data S15

Data S16

## Data Availability

The data that support the findings of this study are available on request from the corresponding author. The data are not publicly available due to privacy or ethical restrictions.

## References

[1] Thurston RC , Joffe H . Vasomotor symptoms and menopause: findings from the Study of Women's Health across the Nation. Obstet Gynecol Clin North Am. 2011 Sep;38(3):489–501. 21961716 10.1016/j.ogc.2011.05.006 PMC3185243

[2] Gold EB , Colvin A , Avis N , Bromberger J , Greendale GA , Powell L , et al. Longitudinal analysis of the association between vasomotor symptoms and race/ethnicity across the menopausal transition: study of women's health across the nation. Am J Public Health. 2006;96(7):1226–35. 16735636 10.2105/AJPH.2005.066936 PMC1483882

[3] Pinkerton JV , Abraham L , Bushmakin AG , Cappelleri JC , Komm BS . Relationship between changes in vasomotor symptoms and changes in menopause‐specific quality of life and sleep parameters. Menopause. 2016;23(10):1060–6. 27404028 10.1097/GME.0000000000000678

[4] Kaunitz AM , Manson JE . Management of menopausal symptoms. Obstet Gynecol. 2015;126(4):859–76. 26348174 10.1097/AOG.0000000000001058 PMC4594172

[5] Avis NE , Crawford SL , Greendale G , Bromberger JT , Everson‐Rose SA , Gold EB , et al. Duration of menopausal vasomotor symptoms over the menopause transition. JAMA Intern Med. 2015;175(4):531–9. 25686030 10.1001/jamainternmed.2014.8063 PMC4433164

[6] Col NF , Guthrie JR , Politi M , Dennerstein L . Duration of vasomotor symptoms in middle‐aged women: a longitudinal study. Menopause. 2009;16(3):453–7. 19188852 10.1097/gme.0b013e31818d414e

[7] Freeman EW , Sammel MD , Lin H , Liu Z , Gracia CR . Duration of menopausal hot flushes and associated risk factors. Obstet Gynecol. 2011;117(5):1095–104. 21508748 10.1097/AOG.0b013e318214f0de PMC3085137

[8] Thurston RC , El Khoudary SR , Tepper PG , Jackson EA , Joffe H , Chen HY , et al. Trajectories of Vasomotor Symptoms and Carotid Intima Media Thickness in the Study of Women's Health Across the Nation. Stroke. 2016;47(1):12–7. 26578657 10.1161/STROKEAHA.115.010600 PMC4696910

[9] Zhu D , Chung HF , Dobson AJ , Pandeya N , Anderson DJ , Kuh D , et al. Vasomotor menopausal symptoms and risk of cardiovascular disease: a pooled analysis of six prospective studies. Am J Obstet Gynecol. 2020;223(6):898.e1–e16. 32585222 10.1016/j.ajog.2020.06.039 PMC7704910

[10] Thurston RC , El Khoudary SR , Sutton‐Tyrrell K , Crandall CJ , Gold EB , Sternfeld B , et al. Vasomotor symptoms and lipid profiles in women transitioning through menopause. Obstet Gynecol. 2012;119(4):753–61. 22433339 10.1097/AOG.0b013e31824a09ec PMC3343636

[11] Thurston RC , Chang Y , Mancuso P , Matthews KA . Adipokines, adiposity, and vasomotor symptoms during the menopause transition: findings from the Study of Women's Health Across the Nation. Fertil Steril. 2013;100(3):793–800. 23755948 10.1016/j.fertnstert.2013.05.005 PMC3759568

[12] Shobeiri F , Jenabi E , Poorolajal J , Hazavehei SM . The association between body mass index and hot flash in midlife women: a meta‐analysis. J Menopausal Med. 2016;22(1):14–9. 27152309 10.6118/jmm.2016.22.1.14 PMC4854655

[13] Gallicchio L , Miller SR , Kiefer J , Greene T , Zacur HA , Flaws JA . Change in body mass index, weight, and hot flashes: a longitudinal analysis from the midlife women's health study. J Womens Health (Larchmt). 2014;23(3):231–7. 24341351 10.1089/jwh.2013.4526 PMC3952652

[14] Thurston RC , Ewing LJ , Low CA , Christie AJ , Levine MD . Behavioral weight loss for the management of menopausal hot flashes: a pilot study. Menopause. 2015;22(1):59–65. 24977456 10.1097/GME.0000000000000274 PMC4270932

[15] Stefan N , Haring HU , Hu FB , Schulze MB . Metabolically healthy obesity: epidemiology, mechanisms, and clinical implications. Lancet Diabetes Endocrinol. 2013;1(2):152–62. 24622321 10.1016/S2213-8587(13)70062-7

[16] Craig CL , Marshall AL , Sjostrom M , Bauman AE , Booth ML , Ainsworth BE , et al. International physical activity questionnaire: 12‐country reliability and validity. Med Sci Sports Exerc. 2003;35(8):1381–95. 12900694 10.1249/01.MSS.0000078924.61453.FB

[17] Harlow SD , Gass M , Hall JE , Lobo R , Maki P , Rebar RW , et al. Executive summary of the Stages of Reproductive Aging Workshop + 10: addressing the unfinished agenda of staging reproductive aging. J Clin Endocrinol Metab. 2012;97(4):1159–68. 22344196 10.1210/jc.2011-3362 PMC3319184

[18] Malavolti M , Mussi C , Poli M , Fantuzzi AL , Salvioli G , Battistini N , et al. Cross‐calibration of eight‐polar bioelectrical impedance analysis versus dual‐energy X‐ray absorptiometry for the assessment of total and appendicular body composition in healthy subjects aged 21‐82 years. Ann Hum Biol. 2003;30(4):380–91. 12881138 10.1080/0301446031000095211

[19] WHO Western Pacific Regionp . The Asia‐Pacific Perspective: Redefining Obesity and Its Treatment. Sydney: Health Communications Australia; 2000.

[20] Alberti KG , Eckel RH , Grundy SM , Zimmet PZ , Cleeman JI , Donato KA , et al. Harmonizing the metabolic syndrome: a joint interim statement of the International Diabetes Federation Task Force on Epidemiology and Prevention; National Heart, Lung, and Blood Institute; American Heart Association; World Heart Federation; International Atherosclerosis Society; and International Association for the Study of Obesity. Circulation. 2009;120(16):1640–5. 19805654 10.1161/CIRCULATIONAHA.109.192644

[21] Matthews DR , Hosker JP , Rudenski AS , Naylor BA , Treacher DF , Turner RC . Homeostasis model assessment: insulin resistance and beta‐cell function from fasting plasma glucose and insulin concentrations in man. Diabetologia. 1985;28(7):412–9. 3899825 10.1007/BF00280883

[22] Chang Y , Kim BK , Yun KE , Cho J , Zhang Y , Rampal S , et al. Metabolically‐healthy obesity and coronary artery calcification. J Am Coll Cardiol. 2014;63(24):2679–86. 24794119 10.1016/j.jacc.2014.03.042

[23] Hilditch JR , Lewis J , Peter A , van Maris B , Ross A , Franssen E , et al. A menopause‐specific quality of life questionnaire: development and psychometric properties. Maturitas. 1996;24(3):161–75. 8844630 10.1016/s0378-5122(96)82006-8

[24] Park JH , Bae SH , Jung YM . Validity and reliability of the Korean version of the menopause‐specific quality of life. J Korean Acad Nurs. 2020;50(3):487–500. 32632080 10.4040/jkan.20049

[25] Barros AJ , Hirakata VN . Alternatives for logistic regression in cross‐sectional studies: an empirical comparison of models that directly estimate the prevalence ratio. BMC Med Res Methodol. 2003;20(3):21. 10.1186/1471-2288-3-21 PMC521200 14567763

[26] Zou G . A modified poisson regression approach to prospective studies with binary data. Am J Epidemiol. 2004;159(7):702–6. 15033648 10.1093/aje/kwh090

[27] Royston P , Parmar MK . Flexible parametric proportional‐hazards and proportional‐odds models for censored survival data, with application to prognostic modelling and estimation of treatment effects. Stat Med. 2002;21(15):2175–97. 12210632 10.1002/sim.1203

[28] Campagnoli C , Morra G , Belforte P , Belforte L , Prelato TL . Climacteric symptoms according to body weight in women of different socio‐economic groups. Maturitas. 1981;3(3–4):279–87. 7334937 10.1016/0378-5122(81)90035-9

[29] Erlik Y , Meldrum DR , Judd HL . Estrogen levels in postmenopausal women with hot flashes. Obstet Gynecol. 1982;59(4):403–7. 7078891

[30] Gold EB , Crawford SL , Shelton JF , Tepper PG , Crandall CJ , Greendale GA , et al. Longitudinal analysis of changes in weight and waist circumference in relation to incident vasomotor symptoms: the Study of Women's Health Across the Nation (SWAN). Menopause. 2017;24(1):9–26. 27749738 10.1097/GME.0000000000000723 PMC5177513

[31] Li J , Liu B , Tang R , Luo M , Li HJ , Peng Y , et al. Relationship between vasomotor symptoms and metabolic syndrome in Chinese middle‐aged women. Climacteric. 2021;24(2):151–6. 33103941 10.1080/13697137.2020.1789094

[32] Ryu KJ , Park HT , Kwon DH , Yang KS , Kim YJ , Yi KW , et al. Vasomotor symptoms and metabolic syndrome in Korean postmenopausal women. Menopause. 2015;22:1239–45. 25899005 10.1097/GME.0000000000000461

[33] Anderson GS . Human morphology and temperature regulation. Int J Biometeorol. 1999;43(3):99–109. 10639901 10.1007/s004840050123

[34] Randolph JF Jr , Sowers M , Bondarenko I , Gold EB , Greendale GA , Bromberger JT , et al. The relationship of longitudinal change in reproductive hormones and vasomotor symptoms during the menopausal transition. J Clin Endocrinol Metab. 2005;90:6106–12. 16144949 10.1210/jc.2005-1374

[35] Derby CA , Crawford SL , Pasternak RC , Sowers M , Sternfeld B , Matthews KA . Lipid changes during the menopause transition in relation to age and weight: the Study of Women's Health Across the Nation. Am J Epidemiol. 2009;169(11):1352–61. 19357323 10.1093/aje/kwp043 PMC2727246

[36] Lo JC , Zhao X , Scuteri A , Brockwell S , Sowers MR . The association of genetic polymorphisms in sex hormone biosynthesis and action with insulin sensitivity and diabetes mellitus in women at midlife. Am J Med. 2006;119(9 Suppl 1):S69–78. 10.1016/j.amjmed.2006.07.009 16949391

[37] Alexander C , Cochran CJ , Gallicchio L , Miller SR , Flaws JA , Zacur H . Serum leptin levels, hormone levels, and hot flashes in midlife women. Fertil Steril. 2010;94(3):1037–43. 19476935 10.1016/j.fertnstert.2009.04.001 PMC3939020

[38] Maury E , Brichard SM . Adipokine dysregulation, adipose tissue inflammation and metabolic syndrome. Mol Cell Endocrinol. 2010;314(1):1–16. 19682539 10.1016/j.mce.2009.07.031

[39] Luheshi GN , Gardner JD , Rushforth DA , Loudon AS , Rothwell NJ . Leptin actions on food intake and body temperature are mediated by IL‐1. Proc Natl Acad Sci U S A. 1999;96(12):7047–52. 10359836 10.1073/pnas.96.12.7047 PMC22051

[40] Leon LR , White AA , Kluger MJ . Role of IL‐6 and TNF in thermoregulation and survival during sepsis in mice. Am J Physiol. 1998;275(1):R269–77. 9688988 10.1152/ajpregu.1998.275.1.R269

[41] Karim R , Dang HM , Hodis HN , Stanczyk FZ , Brinton RD , Mack WJ . Association of hot flushes with ghrelin and adipokines in early versus late postmenopausal women. Menopause. 2020;27(5):512–8. 32049929 10.1097/GME.0000000000001508 PMC7845756

[42] Goossens GH . The Metabolic Phenotype in Obesity: Fat Mass, Body Fat Distribution, and Adipose Tissue Function. Obes Facts. 2017;10(3):207–15. 28564650 10.1159/000471488 PMC5644968

[43] Tuomikoski P , Savolainen‐Peltonen H . Vasomotor symptoms and metabolic syndrome. Maturitas. 2017 Mar;97:61–5. 28159064 10.1016/j.maturitas.2016.12.010

[44] Freedman RR . Menopausal hot flashes: mechanisms, endocrinology, treatment. J Steroid Biochem Mol Biol. 2014;142:115–20. 24012626 10.1016/j.jsbmb.2013.08.010 PMC4612529

[45] Rothman KJ . BMI‐related errors in the measurement of obesity. Int J Obes (Lond). 2008;32(Suppl 3):S56–9. 18695655 10.1038/ijo.2008.87

[46] Sommer I , Teufer B , Szelag M , Nussbaumer‐Streit B , Titscher V , Klerings I , et al. The performance of anthropometric tools to determine obesity: a systematic review and meta‐analysis. Sci Rep. 2020;10(1):12699. 32728050 10.1038/s41598-020-69498-7 PMC7391719

[47] Verweij LM , Terwee CB , Proper KI , Hulshof CTJ , van Mechelen W . Measurement error of waist circumference: gaps in knowledge. Public Health Nutr. 2013;16(2):281–8. 22626254 10.1017/S1368980012002741 PMC10271771

[48] Chung W , Lim SJ , Lee S , Kim R , Kim J . Gender‐specific interactions between education and income in relation to obesity: a cross‐sectional analysis of the Fifth Korea National Health and Nutrition Examination Survey (KNHANES V). BMJ Open. 2017;7:e014276. 10.1136/bmjopen-2016-014276 PMC5770831 29288171

[49] Deurenberg P , Deurenberg‐Yap M , Guricci S . Asians are different from Caucasians and from each other in their body mass index/body fat per cent relationship. Obes Rev. 2002;3(3):141–6. 12164465 10.1046/j.1467-789x.2002.00065.x

