# Supplementary material for: Metabolically healthy and unhealthy obesity and risk of vasomotor symptoms in premenopausal women: cross‐sectional and cohort studies
Source: BJOG. 2022 Jun 7;129(11):1926–34. doi: 10.1111/1471-0528.17224 (PMC9541406; doi:10.1111/1471-0528.17224)
Supplement: Supplementary file 1 — Appendix S1 [file BJO-129-1926-s001.pdf]

Online Supporting Information

Metabolically healthy and unhealthy obesity and risk of vasomotor symptoms in premenopausal women: cross-sectional and cohort studies

Description

Figure S1. Flow diagram for the selection of the study population

Table S1. Baseline characteristics of the study participants by prevalent VMS

Table S2. Baseline characteristics of the study participants by incident VMS

Table S3. Baseline characteristics of the study participants according to follow-up

Table S4. Longitudinal association between adiposity measures and VMS incidence among premenopausal women free of VMS at the baseline based on parametric proportional hazard models using inverse probability weights

Table S5. Longitudinal association between adiposity measures and VMS incidence among premenopausal women free of VMS at the baseline without restriction prior to menopause during follow-up

Table S6. Longitudinal associations between adiposity measures and VMS among metabolically healthy and unhealthy premenopausal women without restriction prior to menopause during follow-up

Table S7. Association between adiposity measures and early-onset vasomotor symptoms among premenopausal women stratified by education level

Figure S2. Multivariable-adjusted prevalence ratios (95% confidence intervals) for early-onset vasomotor symptoms (VMS) using adiposity measures as a continuous factor in a cross-sectional study.

Figure S3. Multivariable-adjusted hazard ratios (95% confidence intervals) for incident early-onset vasomotor symptoms (VMS) using adiposity measures as a continuous factor.

Figure S1. Flow diagram for the selection of the study population

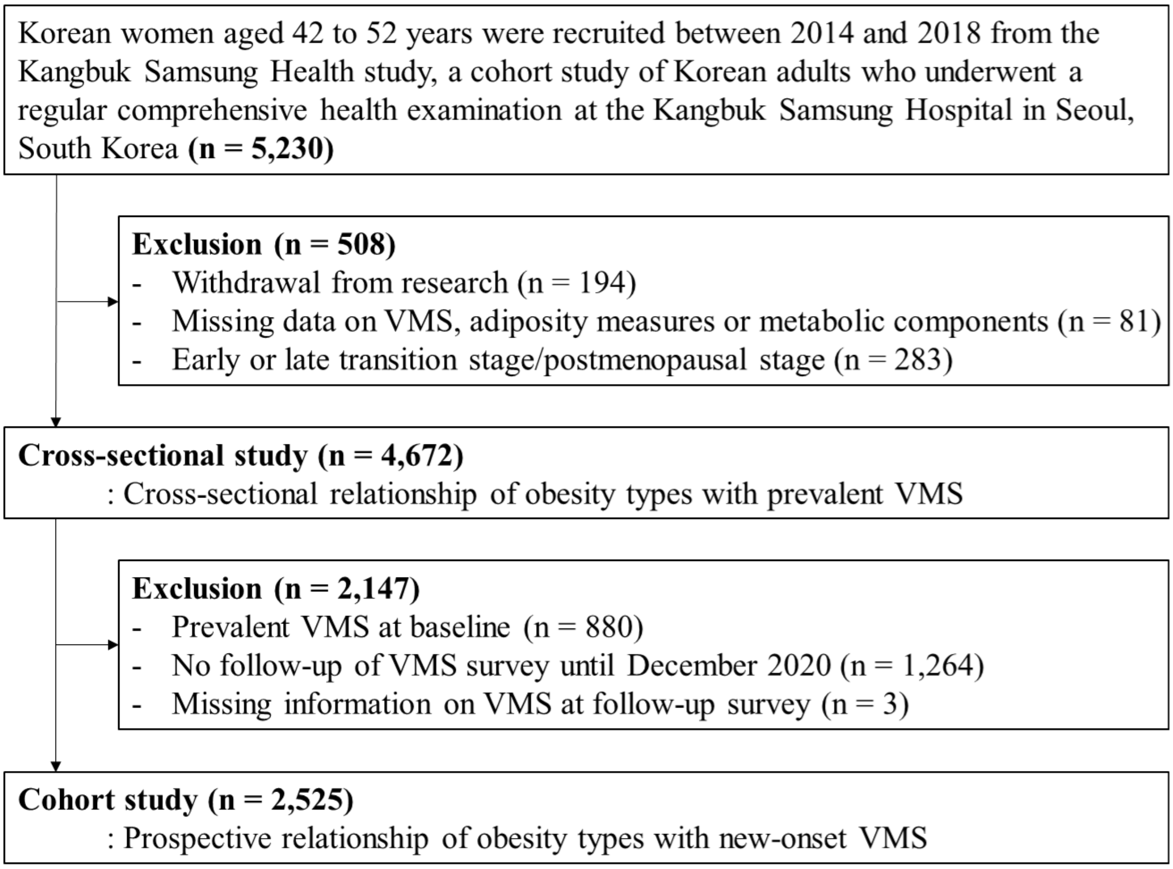

Table S1. Baseline characteristics of the study participants by prevalent VMS

Characteristics	No VMS (n=3,792)	Prevalent VMS (n=880)	P value
Age (y)	44.8±2.4	45.1±2.6	0.03
Being metabolically unhealthy (%)	30.4	40.7	<0.001
Number of metabolic abnormalities			<0.001
0	69.6	59.3	
1	18.8	22.5	
2	7.2	10.9	
≥3	4.4	7.3	
BMI category (kg/m^2)			<0.001
<22.9	65.4	56.4	
23.0-24.9	19.0	21.8	
25.0-29.9	14.0	18.6	
≥30	1.7	3.2	
Waist circumference ≥80 cm (%)	26.6	34.1	<0.001
Percent body fat (%)			<0.001
<25.0	17.7	12.3	
25.0-29.9	30.5	26.5	
30.0-34.9	32.2	33.07	
≥35.0	19.6	28.2	
Age at menarche (y)	14.0±1.4	14.0±1.5	0.728
Parity (%)	92.2	91.1	0.306
Ever smoker (%)	11.5	12.3	0.420
Alcohol intake (%)[a]	12.4	15.4	0.023
HEPA (%)	15.6	15.3	0.116
High education (%)[b]	80.8	77.3	0.022
Hypertension (%)	4.3	7.8	<0.001
Diabetes (%)	1.9	3.0	0.043
Medication for hyperlipidemia (%)	1.5	1.9	0.393
Systolic BP (mmHg)	103.4±11.4	105.7±12.9	<0.001
Diastolic BP (mmHg)	66.6±9.0	68.2±10.0	<0.001
Glucose (mg/dL)	92.9±12.2	94.4±13. 5	0.001
LDL-C (mg/dL)	118.4±28.6	121.4±29.3	<0.001
HDL-C (mg/dL)	67.1±16.0	64.9±15.4	<0.001
Triglycerides (mg/dL)	74.0 (57.0-98.0)	81.0 (61.0-113.0)	<0.001
HOMA-IR	1.1 (0.7-1.6)	1.2 (0.8-1.7)	<0.001
hsCRP (mg/L) [c]	0.03 (0.02-0.1)	0.04 (0.02-0.1)	0.002

Data are presented as the mean ± standard deviation, median (interquartile range), or percentage.
[a]≥10 g of ethanol per day; [b]≥college graduate.
[c] among 3,056 participants
Abbreviations: *ALT*, alanine aminotransferase; *BMI*, body mass index; *BP*, blood pressure; *HDL-C*, high-density lipoprotein cholesterol; *HEPA*, health-enhancing physical activity; *HOMA-IR*, homeostasis model assessment of insulin resistance; *hsCRP*, high-sensitivity C-reactive protein; *LDL-C*, low-density lipoprotein cholesterol.

Table S2. Baseline characteristics of the study participants by incident VMS

Characteristics	No VMS (n=1,693)	Incident VMS (n=832)	P value
Age (y)	44.49±2.27	45.08±2.49	<0.001
Being metabolically unhealthy (%)	28.53	32.57	0.037
Number of metabolic abnormalities			0.014
0	71.47	67.43	
1	18.25	19.71	
2	6.62	7.57	
≥3	3.66	5.29	
BMI category (kg/m^2)			<0.001
<22.9	69.34	59.50	
23.0-24.9	16.42	21.51	
25.0-29.9	12.46	17.19	
≥30	1.77	1.80	
Waist circumference ≥80 cm (%)	23.51	31.01	<0.001
Percent body fat (%)			<0.001
<25.0	19.73	13.58	
25.0-29.9	31.60	29.33	
30.0-34.9	30.66	34.74	
≥35.0	18.02	22.36	
Age at menarche (y)	13.86±1.38	13.98±1.37	
Parity (%)	91.94	92.95	0.383
Ever smoker (%)	11.05	11.66	0.402
Alcohol intake (%)[a]	11.35	11.74	0.775
HEPA (%)	13.93	18.00	0.025
High education (%)[b]	83.58	77.82	<0.001
Hypertension (%)	3.84	4.09	0.764
Diabetes (%)	2.01	1.56	0.436
Medication for hyperlipidemia (%)	1.54	1.56	0.961
Systolic BP (mmHg)	102.86±11.08	104.00±11.62	0.017
Diastolic BP (mmHg)	66.23±8.80	66.61±8.61	0.301
Glucose (mg/dL)	92.44±11.14	93.02±11.09	0.218
LDL-C (mg/dL)	117.16±28.39	119.66±28.43	0.038
HDL-C (mg/dL)	68.0±16.1	66.1±15.7	0.005
Triglycerides (mg/dL)	71.0 (56.0-96.0)	77.0 (58.0-101.5)	0.003
HOMA-IR	1.1 (0.7-1.5)	1.1 (0.8-1.6)	0.102
hsCRP (mg/L) [c]	0.03 (0.02-0.05)	0.03 (0.02-0.06)	0.312

Data are presented as the mean ± standard deviation, median (interquartile range), or percentage.
[a]≥10 g of ethanol per day; [b]≥college graduate.
[c] among 1,727 participants
Abbreviations: *ALT*, alanine aminotransferase; *BMI*, body mass index; *BP*, blood pressure; *HDL-C*, high-density lipoprotein cholesterol; *HEPA*, health-enhancing physical activity; *HOMA-IR*, homeostasis model assessment of insulin resistance; *hsCRP*, high-sensitivity C-reactive protein; *LDL-C*, low-density lipoprotein cholesterol.

Table S3. Baseline characteristics of the study participants according to follow-up

Characteristics	No follow up (n = 1,264)	Follow up (n = 2,525)
Age (y)	45.1±2.6	44.7 ± 2.4
Being metabolically unhealthy (%)	31.5	29.9
Number of metabolic abnormalities		
0	68.5	70.1
1	19.1	18.7
2	7.8	6.9
≥3	4.7	4.2
BMI category (kg/m^2)		
<23	63.9	66.1
23.0-24.9	20.7	18.1
25.0-29.9	13.9	14.0
≥30	1.4	1.8
Waist circumference ≥80 cm (%)	27.9	26.0
Percent body fat (%)		
<25.0	17.6	17.7
25.0-29.9	29.9	30.9
30.0-34.9	32.5	32.0
≥35.0	19.9	19.5
Age at menarche (y)	14.1±1. 5	13.9 ± 1.4
Parity (%)		92.3
Ever smoker (%)	12.0	11.3
Alcohol intake (%)[a]	14.4	11.5
HEPA (%)	16.1	15.3
High education (%)[b]	78.9	81.7
Hypertension (%)	5.1	3.9
Diabetes (%)	1.9	1.9
Medication for hyperlipidemia (%)	1.5	1.6
Systolic BP (mmHg)	103.8±11.5	103.2 ± 11.3
Diastolic BP (mmHg)	67.0±9.4	66.4 ± 8.7
Glucose (mg/dL)	93.4±14.2	92.6 ± 11.1
LDL-C (mg/dL)	119.3±29.0	118.0 ± 28.4
HDL-C (mg/dL)	66.6±15.9	67.4 ± 16.0
Triglycerides (mg/dL)	84.8(58-98)	73 (56─98)
HOMA-IR	1.3 (0.7-1.6)	1.1 (0.8─1.6)
hsCRP (mg/L)	0.3 (0.2-0.6)	0.3 (0.2─0.6)

Data are presented as the mean ± standard deviation, median (interquartile range), or percentage.
[a] ≥10 g of ethanol per day; [b] ≥college graduate.
Abbreviations: *ALT*, alanine aminotransferase; *BMI*, body mass index; *BP*, blood pressure; *HDL-C*, high-density lipoprotein cholesterol; *HEPA*, health-enhancing physical activity; *HOMA-IR*, homeostasis model assessment of insulin resistance; *hsCRP*, high-sensitivity C-reactive protein; *LDL-C*, low-density lipoprotein cholesterol.

Table S4. Longitudinal association between adiposity measures and VMS incidence among premenopausal women free of VMS at the baseline based on parametric proportional hazard models using inverse probability weights

Obesity type	Multivariate-adjusted HR (95% CI)[a]
BMI category (kg/m^2)	
<23.0	Reference
23.0-24.9	1.30 (1.08-1.56)
≥25	1.24 (1.02-1.52)
P for trend	0.007
Waist circumference (cm)	
<80	Reference
≥80	1.22 (1.04-1.43)
Percent body fat (%)	
<25.0	Reference
25.0-29.9	1.23 (0.97-1.56)
30.0-34.9	1.43 (1.14-1.81)
≥35.0	1.52 (1.18-1.96)
P for trend	<0.001
Metabolic health status	
healthy	Reference
unhealthy	1.01 (0.87-1.19)

[a] Parametric proportional hazard models using inverse probability weights were used. The multivariate model was adjusted for age, educational level, parity, physical activity, smoking status, and alcohol intake.

Abbreviations: *BMI*, body mass index; *CI*, confidence interval; *HR*, hazard ratio; *VMS*, vasomotor symptoms.

Table S5. Longitudinal association between adiposity measures and VMS incidence among premenopausal women free of VMS at the baseline without restriction prior to menopause during follow-up (n = 2,677)

Obesity type	Person-years (PY)	Early-onset VMS	Incidence rate (cases per 100 PY)	Age-adjusted HR (95% CI)	Multivariable-adjusted HR (95% CI)[a]
BMI category (kg/m^2)					
<23.0	7312.4	580	7.9	Reference	Reference
23.0-24.9	2035.5	209	10.3	1.28 (1.09-1.51)	1.26 (1.07-1.47)
≥25	1719.4	180	10.5	1.29 (1.08-1.52)	1.28 (1.08-1.52)
P for trend				0.001	0.001
Waist circumference (cm)					
<80	8193.5	669	8.2	Reference	Reference
≥80	2873.9	300	19.4	1.27 (1.11-1.46)	1.27 (1.10-1.46)
Percent body fat (%)					
<25.0	1914.4	131	6.8	Reference	Reference
25.0-29.9	3467.4	282	8.1	1.18 (0.96-1.45)	1.18 (0.96-1.46)
30.0-34.9	3600.8	342	9.4	1.39 (1.13-1.70)	1.39 (1.13-1.70)
≥35.0	2084.9	214	10.3	1.50 (1.20-1.86)	1.52 (1.22-1.89)
P for trend				<0.001	<0.001
Metabolic health status					
healthy	7777.2	655	8.4	Reference	Reference
unhealthy	3290.2	314	9.5	1.11 (0.97-1.27)	1.11 (0.97-1.27)

[a] Parametric proportional hazard models were used. The multivariate model was adjusted for age, educational level, parity, physical activity, smoking status, and alcohol intake.

Abbreviations: *BMI*, body mass index; *CI*, confidence interval; *HR*, hazard ratio; *VMS*, vasomotor symptoms.

Table S6. Longitudinal associations between adiposity measures and VMS among metabolically healthy and unhealthy premenopausal women without restriction prior to menopause during follow-up (n = 2,677)

	Longitudinal analysis (n = 2,677) Multivariable-adjusted hazard ratios (95% CI) [a]		
Adiposity measures	Metabolically healthy (n = 1,873)	Metabolically unhealthy (n = 804)	*P* for interaction
BMI category (kg/m^2)			0.438
<22.9	Reference	Reference	
23.0-24.9	1.23 (1.01-1.50)	1.31 (0.98-1.76)	
25.0-29.9	1.25 (0.96-1.66)	1.28 (1.02-1.80)	
P for trend	0.021	0.057	
Waist circumference (cm)			0.964
<80	Reference	Reference	
≥80	1.29 (1.07-1.56)	1.18 (0.94-1.48)	
Percent body fat (%)			0.011
<25.0	Reference	Reference	
25.0-29.9	1.00 (0.80-1.25)	3.30 (1.69-6.42)	
30.0-34.9	1.28 (1.03-1.60)	2.97 (1.55-5.69)	
≥35.0	1.25 (0.95-1.64)	3.73 (1.95-7.16)	
P for trend	0.012	0.001	

[a] Parametric proportional hazard models were used to estimate the hazard ratios with 95% confidence intervals. The multivariate model was adjusted for age, education level, parity, physical activity, smoking status, and alcohol intake.
Abbreviations: BMI, body mass index; CI, confidence interval; HR, hazard ratio; VMS, vasomotor symptoms.

Table S7. Association between adiposity measures and early-onset vasomotor symptoms among premenopausal women stratified by education level

Obesity type	Multivariable-adjusted Prevalence ratios (95% CI)[b]		*P* for interaction[a]	Multivariable-adjusted Hazard ratios (95% CI)[c]		P for interaction[a]
	Low education (n=911)	High education (n=3,666)		Low education (n=455)	High education (n=2,027)	
BMI category (kg/m²)			0.617			0.770
<23.0	Reference	Reference		Reference	Reference	
23.0-24.9	1.18 (0.84-1.63)	1.25 (1.05-1.48)		1.22 (0.82-1.81)	1.33 (1.10-1.62)	
≥25	1.57 (1.18-2.09)	1.36 (1.14-1.63)		1.42 (0.97-2.06)	1.27 (1.03-1.57)	
P for trend	0.003	<0.001		0.057	0.005	
Waist circumference (cm)			0.806			0.323
<80	Reference	Reference		Reference	Reference	
≥80	1.37 (1.06-1.76)	1.29 (1.11-1.49)		2.68 (2.15-3.33)	1.24 (1.05-1.48)	
Percent body fat (%)			0.289			0.180
<25.0	Reference	Reference		Reference	Reference	
25.0-29.9	0.84 (0.52-1.35)	1.32 (1.04-1.68)		1.00 (0.60-1.69)	1.30 (1.01-1.67)	
30.0-34.9	1.18 (0.77-1.80)	1.42 (1.12-1.79)		1.11 (0.67-1.84)	1.49 (1.16-1.90)	
≥35.0	1.52 (0.98-2.34)	1.84 (1.45-2.34)		1.67 (0.98-2.84)	1.55 (1.18-2.02)	
P for trend	0.004	<0.001		0.029	<0.001	
Metabolic health status			0.291			0.893
healthy	Reference	Reference		Reference	Reference	
unhealthy	1.24 (0.96-1.60)	1.04 (1.00-1.08)		1.01 (0.74-1.38)	1.13 (0.95-1.33)	

[a] P for interaction by education level (less than college graduate vs. college graduate or higher).

[b] Poisson regression models with robust variance were used to estimate prevalence ratios and 95% CIs for prevalent VMS. The multivariate model was adjusted for age, parity, physical activity, smoking status, and alcohol intake.

[c] Parametric proportional hazard models were used to estimate hazard ratios (95% CIs) for incident VMS among premenopausal women without VMS at the baseline. The multivariate model was adjusted for age, parity, physical activity, smoking status, and alcohol intake.

Abbreviations: BMI, body mass index; CI, confidence interval; HR, hazard ratio; VMS, vasomotor symptoms.

Figure S2. Multivariable-adjusted prevalence ratios (95% confidence intervals) for early-onset vasomotor symptoms (VMS) using adiposity measures as a continuous factor in a cross-sectional study. The curves represent adjusted hazard ratios (solid line) and their 95% confidence intervals (dashed lines) for incident early-onset VMS on the basis of restricted cubic splines for each adiposity measure ([a] BMI, [b] waist circumference, and [c] body fat percentage with knots at the 5th, 27.5th, 50th, 72.5th, and 95th percentiles). The model was adjusted for age, educational level, parity, physical activity, smoking status, and alcohol intake.

Abbreviations: BMI, body mass index; VMS, vasomotor symptoms

(a) BMI

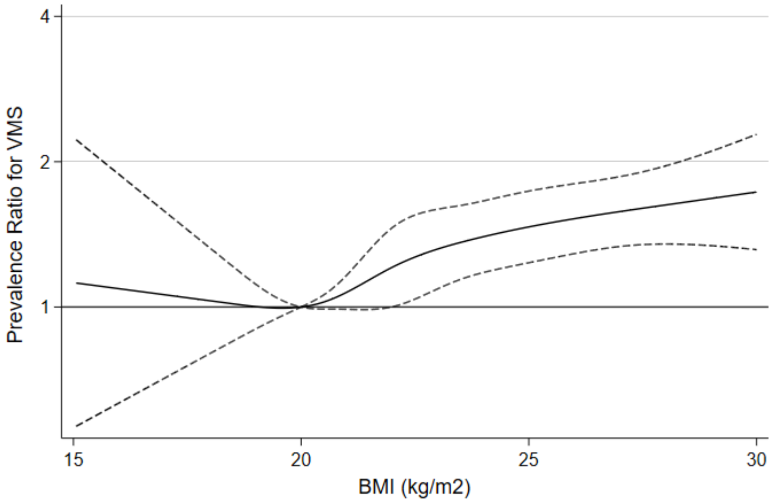

(b) Waist circumference

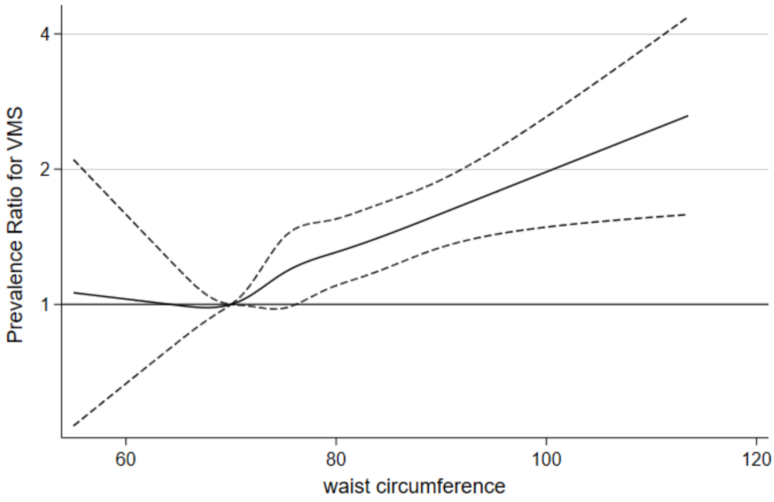

(c) Body fat percentage

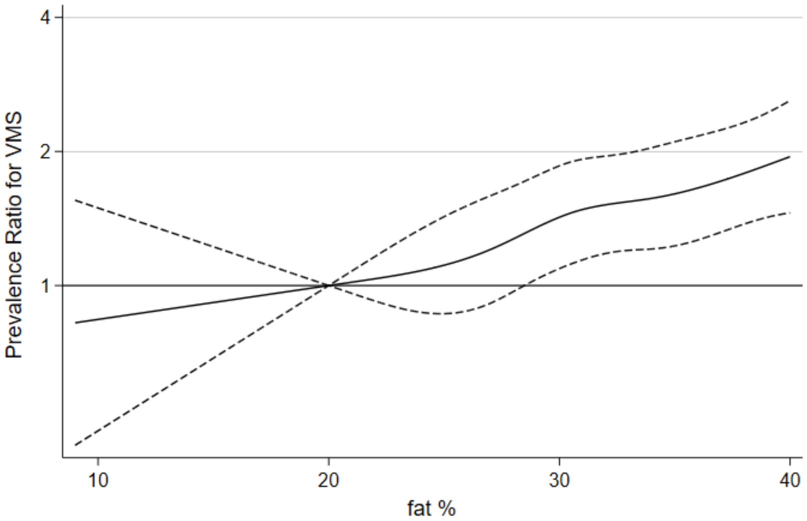
Prevalence Ratio for VMS
4
2
1
10
20
30
40
fat %

Figure S3. Multivariable-adjusted hazard ratios (95% confidence intervals) for incident early-onset vasomotor symptoms (VMS) using adiposity measures as a continuous factor. The curves represent adjusted hazard ratios (solid line) and their 95% confidence intervals (dashed lines) for incident early-onset VMS on the basis of restricted cubic splines for each adiposity measure ([a] BMI, [b] waist circumference, and [c] body fat percentage with knots at the 5th, 27.5th, 50th, 72.5th, and 95th percentiles). The model was adjusted for age, educational level, parity, physical activity, smoking status, and alcohol intake.

Abbreviations: BMI, body mass index; VMS, vasomotor symptoms

(a) BMI

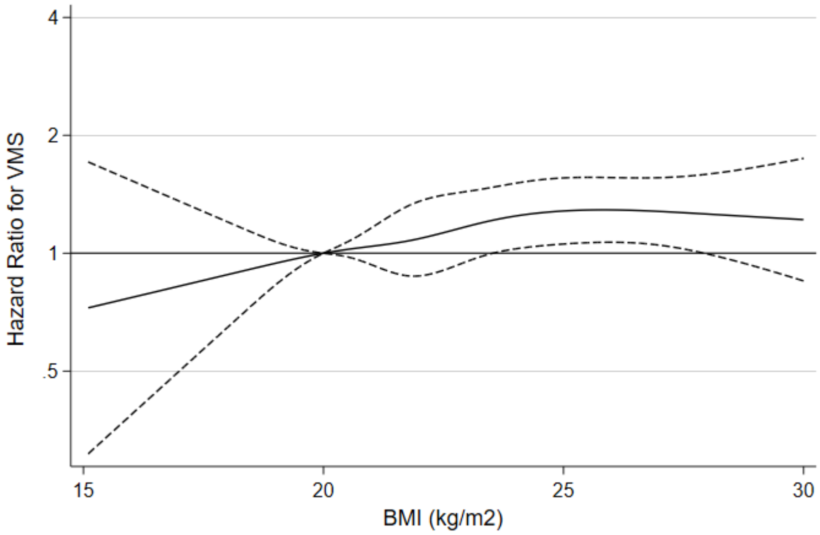

(b) Waist circumference

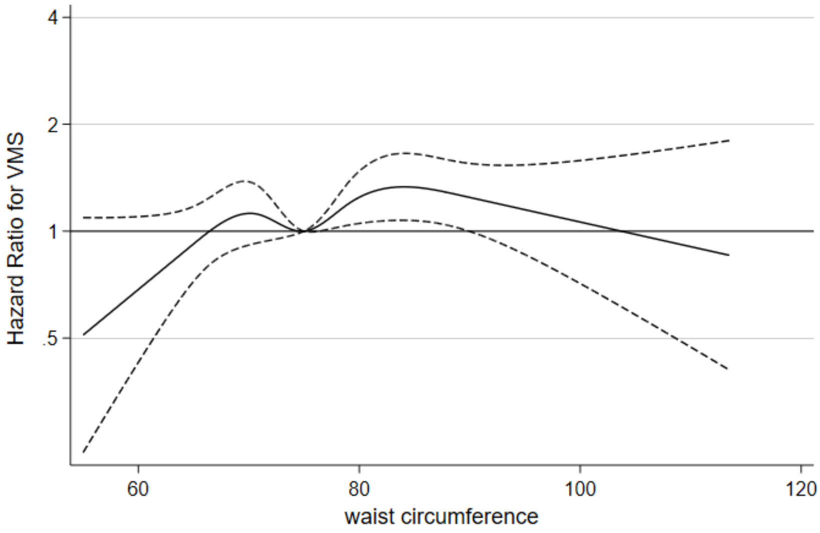

(c) Body fat percentage

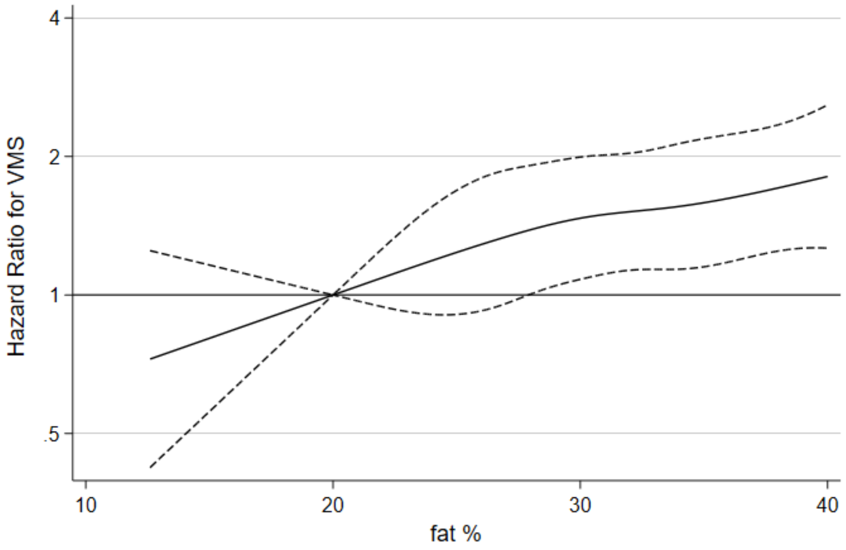

4
2
1
.5
Hazard Ratio for VMS
10
20
30
40
fat %
